# Using Population and Comparative Genomics to Understand the Genetic Basis of Effector-Driven Fungal Pathogen Evolution

**DOI:** 10.3389/fpls.2017.00119

**Published:** 2017-02-03

**Authors:** Clémence Plissonneau, Juliana Benevenuto, Norfarhan Mohd-Assaad, Simone Fouché, Fanny E. Hartmann, Daniel Croll

**Affiliations:** ^1^Plant Pathology, Institute of Integrative Biology, ETH ZurichZurich, Switzerland; ^2^UMR, BIOGER, INRA, AgroParisTech, Université Paris-SaclayThiverval-Grignon, France; ^3^College of Agriculture “Luiz de Queiroz”, University of São PauloSão Paulo, Brazil; ^4^School of Biosciences and Biotechnology, Faculty of Science and Technology, Universiti Kebangsaan MalaysiaSelangor, Malaysia; ^5^Laboratory of Evolutionary Genetics, Institute of Biology, University of NeuchatelNeuchatel, Switzerland

**Keywords:** plant pathogens, genome, population genomics, evolution, molecular, fungi, agricultural ecosystems, association mapping studies

## Abstract

Epidemics caused by fungal plant pathogens pose a major threat to agro-ecosystems and impact global food security. High-throughput sequencing enabled major advances in understanding how pathogens cause disease on crops. Hundreds of fungal genomes are now available and analyzing these genomes highlighted the key role of effector genes in disease. Effectors are small secreted proteins that enhance infection by manipulating host metabolism. Fungal genomes carry 100s of putative effector genes, but the lack of homology among effector genes, even for closely related species, challenges evolutionary and functional analyses. Furthermore, effector genes are often found in rapidly evolving chromosome compartments which are difficult to assemble. We review how population and comparative genomics toolsets can be combined to address these challenges. We highlight studies that associated genome-scale polymorphisms with pathogen lifestyles and adaptation to different environments. We show how genome-wide association studies can be used to identify effectors and other pathogenicity-related genes underlying rapid adaptation. We also discuss how the compartmentalization of fungal genomes into core and accessory regions shapes the evolution of effector genes. We argue that an understanding of genome evolution provides important insight into the trajectory of host-pathogen co-evolution.

## Introduction

Fungal plant pathogens are major threats to modern agricultural ecosystems and global food security (Strange and Scott, 2005). Many pathogen populations have a high evolutionary potential that enables a rapid response to selection pressure. A better understanding of the processes driving rapid pathogen evolution may be useful for designing more efficient and durable disease management strategies. A dramatic consequence of pathogen evolution is the rapid breakdown of host resistance observed repeatedly in many crops and over many decades (Brown, 2015). Pathogens surmount plant resistance by secreting effector proteins that manipulate the immune system and host metabolism. These effector genes evolve quickly and usually lack homologs in closely related species. Most effector genes encode small, cysteine-rich proteins that are secreted and highly expressed *in planta* (Lo Presti et al., 2015). In response, the host evolved an immune system based primarily on R genes encoding proteins that can directly or indirectly recognize effectors, and trigger defense mechanisms (Jones and Dangl, 2006). This genetic interaction between an avirulence effector (Avr) and its cognate resistance protein is known as the gene-for-gene model (GFG) (Flor, 1971).

The recent broad accessibility of genome sequencing technologies enabled genome-scale analyses of a rapidly growing number of plant pathogen species. These analyses revealed major factors contributing to disease emergence and made plant pathogenic fungi some of the best-studied models of pathogen evolution. Highlights of studies on plant pathogen genomes include the highly plastic genome structure, repertoires of 100s of rapidly evolving candidate effector genes and the non-random distribution of effector genes in the genome. Intriguingly, effector genes are preferentially located in repeat-rich and gene-poor regions of the genome (Raffaele and Kamoun, 2012). The compartmentalization of the genome (i.e., the “two-speed” genome) and the preferential location of effector genes in rapidly evolving compartments emerged independently several times in different fungal lineages and likely plays a key role in the ability of plant pathogens to rapidly respond to selection (Dong et al., 2015).

**FIGURE 1:**
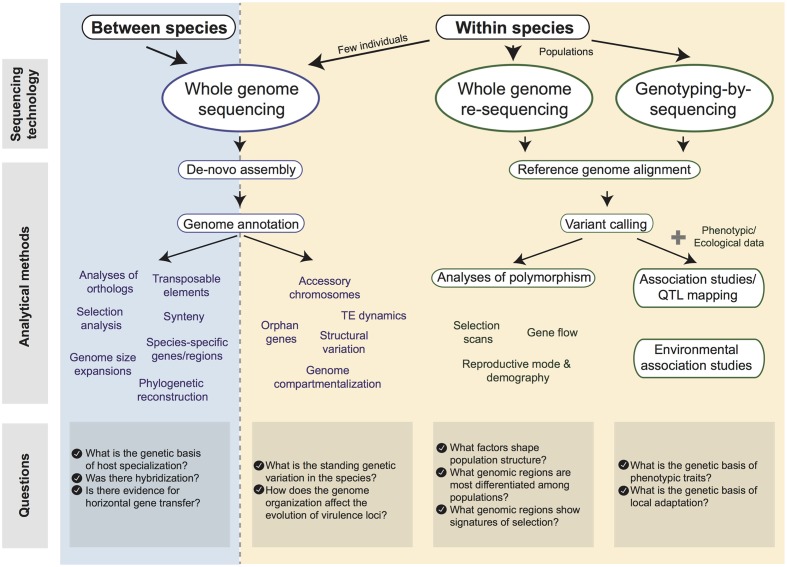
**Diagram of tools available for the analysis of fungal pathogen genomes.** We distinguish “between-species” (blue) and “within-species” (yellow) datasets. Available sequencing technologies and possible analytical methods are shown. Major research questions associated with each type of dataset are shown at the bottom.

The genomic revolution also provides a major opportunity to bridge the gaps between molecular biology, evolutionary genetics and epidemiology. Here, we review the major advances made possible by comparative and population genomics to better understand the biology and evolution of effectors. We focus particularly on the assembly of multiple reference genomes and association mapping, which have the potential to significantly accelerate our understanding of pathogen biology (**Figure 1**). We also discuss the fascinating role accessory genomic regions play in the evolution of effector genes.

### What Makes a Pathogen: Comparative Genomics of Fungal Lineages

The ability to cause disease on plants is a widely shared trait in the fungal kingdom. However, plant pathogenic lineages arose many times independently during fungal evolution (James et al., 2006; Soanes et al., 2007). Hence, different lineages of plant pathogens likely evolved the ability to cause disease through different mechanisms. To shed light on processes governing the evolution of pathogenicity, comparative genomics tools were applied widely across the fungal kingdom. Critical first steps in comparative genomic analyses are generating high-quality genome assemblies (Gibriel et al., 2016). A finished genome assembly (i.e., a gapless assembly from telomere to telomere) enables analyses of synteny breakpoints between homologous chromosomes of closely related species and ensures that the gene content of a genome is accurately reflected in genome annotations. Accurate gene predictions are particularly important to identify evolutionary novelty such as recently emerged effector genes.

**FIGURE 2:**
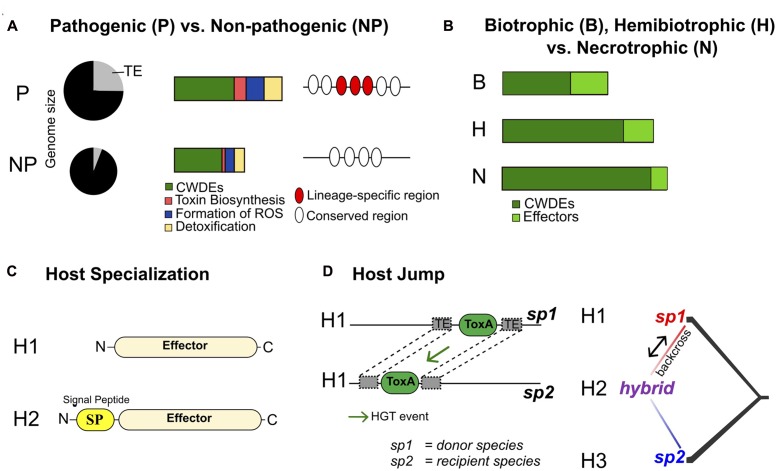
**The genomic basis of pathogenicity, lifestyle and host adaptation in plant pathogens.** We highlight four major types of comparative genomics analyses used to identify unique characteristics of pathogen lineages: **(A)** Comparisons of the genome content of closely related pathogenic (P) vs. non-pathogenic (NP) lineages. Examples of genome expansion due to transposable elements, differences in gene composition and lineage-specific regions are shown. **(B)** Broad comparisons of unrelated fungi with different lifestyles. Specific lifestyles may be reflected in the number of genes in specific functional categories (e.g., effectors and cell wall degrading enzymes, CWDE). **(C)** The evolution of host specialization driven by changes in effector genes. Two stages of effector evolution involving gain of a secretion signal are shown. The gain of a secreted effector enables the pathogen to manipulate a new host (H2). **(D)** A pathogen host jump enabled by a horizontal gene transfer (e.g., ToxA). The recipient strain gains virulence on the same host (H1) that the donor strain is able to infect. Emergence of a pathogen on a new host (H2) through hybridization of two pathogen lineages infecting hosts H1 and H3. See the main text for references documenting each of these scenarios.

Saprophytic and necrotrophic fungi feed on dead plant tissues, while biotrophic and endophytic species require living plant cells as a nutrient source. Hence, different fungal species evolved distinct sets of genes to successfully complete their infection lifestyle. Comparative genomics is a powerful tool to decipher specific gene completements associated with fungal lifestyles. Lo Presti et al. (2015) analyzed the genomes of 89 fungal species exhibiting different lifestyles and found that genomes of saprotrophs, necrotrophs, and hemibiotrophs were significantly enriched in plant cell wall degrading enzymes (PCWDE). In contrast, genomes of obligate biotrophs and symbionts were enriched in small secreted proteins (**Figure 2**). Among plant pathogens, biotrophic fungi have a higher proportion of species-specific predicted effectors than hemibiotrophic and necrotrophic fungi (Kim et al., 2016). Furthermore, biotrophic fungi tend to have fewer carbohydrate-active enzymes (CAZymes) than necrotrophic and hemibiotrophic fungi (Zhao et al., 2014; Kim et al., 2016). Duplessis et al. (2011) compared the genomes of two ascomycetes and 10 basidiomycetes, with a focus on the two obligate biotroph rust species *Melampsora larici-populina* and *Puccinia graminis* f. sp. *tritici*, the causing agents of poplar leaf rust and wheat and barley stem rust, respectively. The genomes of these obligate pathogens are among the largest fungal genomes. Genome expansions were caused by the proliferation of transposable elements and large gene families including oligopeptide and amino acid transporters, copper/zinc superoxide dismutase, helicases, kinases and transcription factors. Lineage-specific expansions included genes encoding small secreted proteins. These putative effectors were highly up-regulated *in planta* and may play a role in host specialization. The obligate biotrophic lifestyle of the two analyzed rust species was associated with a reduction in polysaccharide degrading enzymes and deficiencies in nitrate and sulfate assimilation pathways.

The classification of fungal species into a clear infection lifestyle category can be challenging. For example, *Zymoseptoria tritici* was long considered to have both biotropic and nectrophic lifestyles (hemibiotrophic). Recently, *Z. tricitic* was proposed to be primarily a nectroph with an early latency period (Sánchez-Vallet et al., 2015a). Similarly, *Ramularia colo-cygni* was long described as an asymptomatic endophyte of barley, but recent studies showed that the fungus can induce necrotic lesions and leaf senescence. Hence, *R. colo-cygni* is now considered to be an emerging pathogen of barley (Walters et al., 2008).

Comparing the gene inventory of fungal plant pathogen genomes can also provide insights into conserved pathogenicity mechanisms and lineage-specific innovations (**Figure 2**). Soanes et al. (2008) compared the sets of predicted proteins in the genomes of 34 pathogenic and non-pathogenic fungi and oomycetes. The study found that plant pathogenic species shared no unique sets of genes compared to non-pathogens. This suggests that universal pathogenicity factors may be rare. Nevertheless, protein families potentially involved in pathogenic processes, such as plant cell wall degradation, toxin biosynthesis, formation of reactive oxygen species and detoxification were expanded in the genomes of pathogens (Soanes et al., 2008).

Many genes involved in host–pathogen interactions are under continuous selection pressure which leaves footprints of selection in both the pathogen and host genomes (Aguileta et al., 2009). A classical test for the detection of positive selection is based on the ratio of non-synonymous (dN) to synonymous (dS) substitution rates. The dN/dS ratio indicates the direction and magnitude of selection (purifying and positive selection). Since plant pathogenic fungi are involved in an “arms race” with their host plant, genes involved in virulence are likely to show marks of positive selection. For example, in *Puccinia graminis*, selection analyses showed that pathogen-associated gene families were highly polymorphic and almost the only families under positive selection (Sperschneider et al., 2014; Upadhyaya et al., 2015). In *Z. tritici*, genes involved in adaptation to wheat were identified based on comparisons against two closely related sister species infecting wild grasses (Stukenbrock et al., 2011). A total of 27 positively selected genes were identified, none of them having a predicted function. However, two of those genes were shown to play a role in virulence (Poppe et al., 2015). Analyses of selection among different species often fail to identify signatures of recent, strong selection (e.g., selection imposed by a new host or a new fungicide). Intra-specific analyses of selection (i.e., based on populations) require different methodologies and are discussed below.

A series of recent studies showed that hybridization can lead to the emergence of new plant pathogens (**Figure 2**; Stukenbrock, 2016). Menardo et al. (2016) sequenced and analyzed the genomes of 46 isolates of *Blumeria graminis* from four different *formae speciales* causing powdery mildew diseases on wheat (*B.g. tritici*), durum wheat (*B.g. diccoci*), rye (*B.g. secalis*), and triticale (*B.g. triticale*) which is a hybrid between wheat and rye. The authors showed that a hybridization event between the two *formae speciales B.g. tritici* and *B.g. secalis* gave rise to the *B.g. triticale* adapted to triticale. Genome-wide scans for nucleotide diversity among *B.g. triticale* isolates showed that large chromosomal tracts were either nearly identical or highly diverse. In contrast, nucleotide diversity was monotonic among isolates of other *formae speciales*. The study by Menardo et al. (2016) provides a fascinating example of “mirrored” evolution in the host (which originated from hybridization between wheat and rye) and the pathogen (which originated from hybridization between *B.g. tritici* and *B.g. secalis*). An hybridization event between two distinct lineages of *Z. tritici* (Stukenbrock et al., 2012) is also responsible for the emergence of the species *Z. pseudotritici*. The hybridization event occurred approximately 380 sexual generations ago. These examples highlight the role hybridization events can play in the emergence of pathogens.

### Catching Evolution in Action: The Population Genomics of Plant Pathogens

While comparative genomics provides powerful insight into the evolution of pathogen lineages, findings from comparative genomics studies are often limited to evolutionary events from the distant past. Agricultural pathogens evolve major phenotypes over much shorter time periods. In modern agricultural ecosystems, the breakdown of host resistance often happens within only a few years (Brown, 2015). For example, *Leptosphaeria maculans*, the causal agent of stem canker on oilseed rape, overcame resistance conferred by the gene *Rlm1* within 5 years after its introduction into new cultivars (Rouxel et al., 2003). The evolutionary history and demography of pathogen populations play key roles in the ability of a pathogen to rapidly respond to selection. For an example, population genomics analyses identified major changes in the population structure of *Puccinia striiformis* f. sp. *tritici* over recent years. The population structure changes were accompanied by changes in virulence (Hubbard et al., 2015). Hence, investigating the population structure is an important step in identifying the pathogen’s evolutionary potential.

The ability of the pathogen to disperse varies among plant pathogens and is reflected in the population structure and levels of gene flow (McDonald and Linde, 2002; Zhan et al., 2009). In *Fusarium graminearum*, the causal agent of Fusarium head blight on wheat, barley and maize, natural field populations from different regions in Germany showed low differentiation, suggesting a high gene flow at the regional scale (Talas and McDonald, 2015). In contrast, a study found high differentiation among *Phytophtora infestans* populations infecting two different hosts (cultivated potatoes and wild *Solanum* spp.) despite being collected in the same valley in Mexico. Incipient host specialization among pathogen populations and genetic drift could explain such population subdivision (Flier et al., 2003).

The mode of reproduction also has an impact on the population structure and genotypic diversity (McDonald and Linde, 2002). High recombination rates reduce linkage disequilibrium in the genome. Populations of the sexually reproducing wheat pathogen *Z. tritici* were shown to be genetically diverse and exhibit strong signatures of recombination (Linde et al., 2002; Croll et al., 2015). In contrast, asexual reproduction leads to clonal populations with low genetic diversity. *Fusarium oxysporum* formae speciales are distinct asexual clonal lineages that are often host specialized. *F. oxysporum* genomes harbor both highly conserved genomic regions and lineage-specific genomic regions that lack evidence of recombination (Ma et al., 2010). The existence of asexual fungal lineages recently led to debates about the evidence of sex and how fungal lineages could cope without it (Seidl and Thomma, 2014). One recent example is *Verticillium dahliae*, which causes vascular wilt on a variety of different hosts and may have evolved in the absence of sex. Population genomic studies provided evidence for cryptic sexual reproduction among clonal lineages despite strong indications for long-term clonality (de Jonge et al., 2013; Milgroom et al., 2014).

### Using Genome Scans to Identify Adaptive Loci in Pathogen Genomes

**FIGURE 3:**
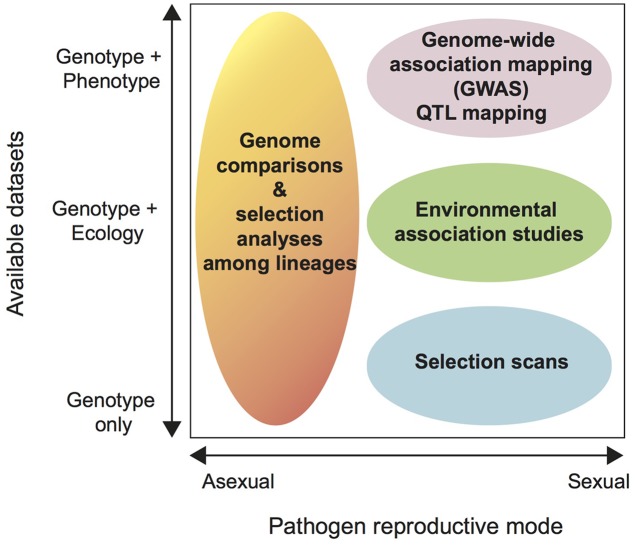
**Decision diagram to determine suitable analysis strategies for intra-specific genomic datasets.** The horizontal axis shows the dominant reproductive mode of the pathogen ranging from completely asexual to sexual. The vertical axis shows the available datasets for the pathogen from “genotype only” to “genotype + ecology” to “genotype + phenotype.” By “ecology” we mean environmental data available for each of the sampled pathogen populations, including abiotic factors such as annual mean temperature, humidity, UV radiation and biotic factors such as the host genotype or the presence or absence of competitors. Phenotype includes any phenotypic data collected from each pathogen strain. Phenotypes are ideally assessed in common-garden settings (e.g., in the same greenhouse compartment or Petri dish) to avoid confounding environmental factors. We discuss each available analysis strategy in the main text.

Among the most powerful applications of population genomics are genome-wide scans of selection. Until now, most intra-specific analyses of selection were restricted to analyses of non-synonymous substitutions in effector genes (e.g., Schürch et al., 2004; Gout et al., 2007; Stukenbrock and Mcdonald, 2007, Stukenbrock and McDonald, 2009). These types of selection analyses assume absence of intra-genic recombination and are most suitable for genes with substantial divergence (see above). In contrast, genome-wide selection scans search for shifts in allele frequencies consistent with a selective sweep, which are much earlier signatures of selection than non-synonymous substitutions. It is important to note that genome-wide selection scans are only as powerful as signatures of selection can be robustly distinguished from genetic drift (Terauchi and Yoshida, 2010; Grünwald et al., 2016). Genome scans aim to identify signatures of selection contributing to ecological adaptation without relying on phenotypes (**Figure 3**). When genome scans are performed across populations spanning an ecological gradient the genome scans can be utilized for “reverse ecology,” ecological genomics or landscape genomics (**Figure 3**). Genome scans test individual loci across the genome for significant departures from neutrality (Oleksyk et al., 2010; Terauchi and Yoshida, 2010; Savolainen et al., 2013). Environmental association studies are increasingly performed in plant species (Weigel and Nordborg, 2015). Striking findings include allele frequencies correlated with climate factors and the finding of candidate genes for climate adaptation in *Arabidopsis thaliana* (Hancock et al., 2011; Fischer et al., 2013). To our knowledge, environmental association studies have not yet been applied to any fungal plant pathogens. A frequently used method for genome scans is to calculate fixation indices (e.g., Wright’s F_ST_) to identify regions of unusual differentiation among populations. F_ST_ outliers are likely to result from divergent selection due to local adaptation. In the model species *Neurospora crassa*, Ellison et al. (2011) used this approach to identify genomic islands of divergence between two subtropical populations. These islands of divergence were hypothesized to stem from adaptation to different temperature and daylight exposures. Functional characterization of candidate genes located in the genomic islands confirmed the role of these genes in temperature adaptation (Ellison et al., 2011). Branco et al. (2015) recently used a similar approach to compare a coastal and a mountain population of the ectomycorrhizal fungus *Suillus brevipes* to investigate the genetic basis of adaptation to saline environments. Population divergence was found to be restricted to a few genomic regions and one of these regions contained a gene known to enhance salt tolerance in plants and yeast (Branco et al., 2015). Ideally, a continuous sampling of natural populations should be made across geographical or ecological gradients. For studies on plant pathogens, gradients could include, e.g., temperature, precipitation, and latitude. However, categorical differences in environments can also be used (e.g., different host genotypes).

Positive selection causes a beneficial allele to increase in frequency in a population. Because most alleles are in linkage disequilibrium with alleles at nearby loci, selection on one allele can affect an entire genomic region, an effect called hitchhiking. Strong selection can lead to a selective sweep that depletes genetic diversity in the nearby genomic region. Sweeps are called hard (i.e., a single haplotype rises to high frequency) or soft (i.e., multiple haplotypes are selected simultaneously) depending on the strength of selection and the amount of standing genetic variation. Site frequency spectrum-based tests, including the commonly used Tajima’s D test, aim to quantify the skew toward rare alleles. Linkage disequilibrium-based methods are used to identify regions with extended haplotypes (Nielsen et al., 2005; Vitti et al., 2013). Selective sweep scans led to identification of loci involved in high-altitude adaptation of humans (Fu and Akey, 2013; Bigham and Lee, 2014) and local adaptation in *A. thaliana* (Long et al., 2013; Huber et al., 2014).

Selection scans are increasingly applied to non-model organisms and are often combined with environmental association studies (Evans et al., 2014; Baute et al., 2015; Bonhomme et al., 2015; Weigel and Nordborg, 2015). Such genome scans are also applicable to plant pathogens (see e.g., Terauchi and Yoshida, 2010; Skrede and Brandström Durling, 2015; Grünwald et al., 2016). A major application will be to investigate the genetic basis of host adaptation. Heterogeneity in the deployment of resistant cultivars over various spatial scales is expected to lead to local adaptation that may be detected by genome scans. For example, adaptation to specific wheat cultivars was found in *Z. tritici* populations sampled from different cultivars in the same field (Cowger et al., 2000). Hence, genome scans could be used to investigate the signatures of adaptation to different hosts or climatic conditions. The ability to hierarchically sample pathogen populations across fields, regions and continents should facilitate such analyses.

Despite their obvious potential, genome scans may not be applicable to all species or may be constrained by confounding factors (**Figure 3**; Levy and Borenstein, 2013; Savolainen et al., 2013). For example, to ascertain the role of specific loci in adaptation, genome scans must be combined with trait mapping or functional characterization (Stinchcombe and Hoekstra, 2008). In addition, strong population differentiation and significant clonal reproduction can significantly affect the power of genome scans. This is because the degree of association among alleles in a population (i.e., linkage disequilibrium) has a strong impact on the ability to detect signatures of selection (Terauchi and Yoshida, 2010; Jensen et al., 2016). High recombination rates may impede the detection of selective sweeps by eroding haplotype block structures in regions under selection. At the same time, high recombination rates may increase the resolution of genome scans by narrowing candidate regions while low recombination rates increase linkage disequilibrium in populations and weaken the ability of selection to favor particular alleles. Hence, highly clonal populations (e.g., of the rice blast pathogen *Pyricularia oryzae* or the potato late blight pathogen *P. infestans*) are unlikely to show strong locus-specific signatures of selection. Selection in mainly asexual pathogens is more likely to act at the level of individual clones than individual alleles. Selection detection methods based on divergence (e.g., dN/dS ratios) are more suitable for largely clonal organisms (Shapiro et al., 2009).

### Association Mapping Techniques to Identify Fungal Virulence Genes

Research over the past decades showed that GFG interactions govern many host–plant pathogen interactions, however, identifying the effector genes involved in the interaction is still challenging. Computational approaches can be used to rapidly screen genomes for candidate effectors. However, such screens lack discriminatory power due to our incomplete understanding of the fungal effector delivery system (Sperschneider et al., 2015, 2016). Furthermore, many candidate effectors were found to have no discernible impact on the host physiology and, hence, may either not be interacting with the host, have a redundant function or their impact is too weak to be quantifiable. Association mapping techniques based on natural variation in virulence are promising to provide a powerful complement to *in silico* predictions of effectors.

**FIGURE 4:**
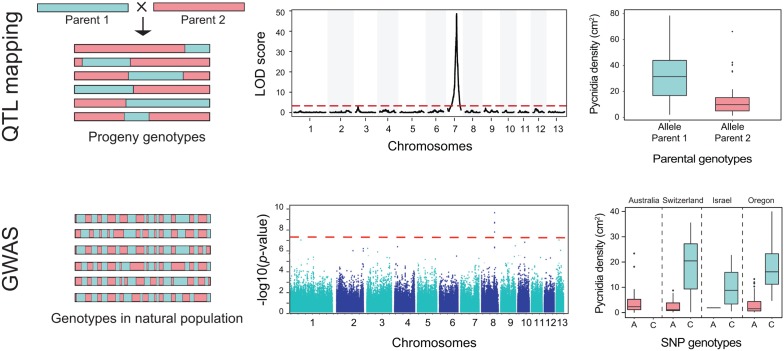
**Identifying genes underlying phenotypic traits using quantitative trait locus (QTL) mapping and genome-wide association studies (GWAS).** The first row illustrates QTL mapping and the second row illustrates GWAS. Genotypes in QTL mapping populations are fundamentally different from genotypes in GWAS populations. The horizontal blocks represent chromosomes from different strains. Chromosomes in QTL mapping are composed of large blocks inherited from the different parents. The block length is determined by the amount of recombination between the parental chromosomes. In contrast, chromosomes in field populations underwent numerous rounds of recombination. Due to this, linkage disequilibrium decays rapidly in GWAS mapping populations. Due to this, GWAS has generally a much stronger ability to resolve associations to small chromosomal regions. The second row shows the result of a QTL mapping with a QTL with a high logarithm of the odds (LOD) ratio located on chromosome 7. The red dotted line shows the significance threshold. The Manhattan plot below shows a significant association on chromosome 8 above the Bonferroni significance threshold. The third row shows the association of phenotypes and genotypes at a significant QTL and a GWAS SNP. The GWAS included populations from four different countries. The phenotype shown is the number of asexual fruiting bodies (pycnidia) per leaf area. Datasets shown here are from Hartmann et al. (2016) and Stewart et al. (2016).

While selection scans aim to identify loci under selection without prior knowledge of associated phenotypes, quantitative trait locus mapping (QTL) and genome-wide association studies (GWAS) are designed to identify loci that mediate heritable phenotypic variation. GWAS and QTL differ primarily in the selection of the mapping populations (**Figure 4**). QTL mapping and GWAS can be complementary approaches with complementary strengths. QTL mapping analyzes genetic variants segregating among progenies of a cross, whereas GWAS utilizes genetic variation among unrelated individuals from a natural population. Both GWAS and QTL mapping aim to associate polymorphism at loci with variation for a measured phenotypic trait. QTL mapping is only possible in pathogens for which crosses can be performed. While GWAS does not have this limitation, the power to map a phenotypic trait to a single locus is directly correlated with the level of recombination in a population. Extensive clonal reproduction leads to linkage disequilibrium across the genome and requires correction prior to association mapping analyses. QTL mapping is more reductionist as only phenotypic and genotypic variation existing between the parents can be mapped. GWAS populations generally exhibit a broader range of phenotypes and genotypes that can be screened. Furthermore, genotypes from field populations of sexual pathogens are generally much more recombined than progeny in a cross, which can dramatically increase the physical resolution of associations (**Figure 4**; Korte and Farlow, 2013; Grünwald et al., 2016).

The genotyping of a mapping population can be based on whole-genome sequencing or reduced-representation sequencing such as restriction-site associated DNA sequencing (RADseq) and genotyping-by-sequencing (GBS). RADseq and GBS offer excellent QTL mapping resolution at low cost (Davey and Blaxter, 2010; Elshire et al., 2011). RADseq and GBS methods reduce the number of loci to be sequenced in the genome by sequencing only at predefined restriction sites. Hence, the choice of restriction enzymes should be carefully evaluated to optimize marker density and sequencing costs (Leboldus et al., 2015). Both RADseq and GBS were successfully used for QTL mapping and GWAS analyses in fungi (Lendenmann et al., 2014, 2015, 2016; Leboldus et al., 2015; Gao et al., 2016; Talas et al., 2016). Nevertheless, the use of reduced-representation sequencing for GWAS should be carefully evaluated. The key parameter to consider is the distance at which linkage disequilibrium decays in the population. If the average distance between markers exceeds the average distance at which linkage disequilibrium decays to low levels, there is a significant risk of false negatives (i.e., missing a region in the genome associated with a phenotype).

Quantitative trait locus mapping has been widely used to identify chromosomal regions linked to phenotypic variation between parents. However, the limited amount of recombination in a single cross retains large linkage blocks that can limit mapping resolution (**Figure 4**; Hall et al., 2010; Dalman et al., 2013; Korte and Farlow, 2013). Therefore, loci identified by QTL mapping are typically large genomic regions with a high number of candidate genes. Another disadvantage of QTL mapping is the limitation to polymorphism segregating between parental isolates (Hall et al., 2010). Hence, additional sets of crosses or generating multi-parental mapping populations are desirable (Kover et al., 2009; Korte and Farlow, 2013).

Quantitative trait locus mapping has rarely been performed in fungi compared to plants. The few fungal QTL studies were nevertheless highly successful. Virulence loci in several fungal species including *Heterobasidion annosum sensu lato, F. graminearum* and *Z. tritici* were mapped using RAPD and AFLP markers (Kema et al., 2002; Cumagun et al., 2004; Lind et al., 2007). More recently, Christians et al. (2011) used whole-genome sequencing to genotype progenies of an *Aspergillus nidulans* cross and mapped three QTLs affecting *in vivo* virulence. Stewart et al. (2016) identified a major locus in *Z. tritici* governing virulence on a specific wheat cultivar by performing QTL mapping of virulence phenotypes measured with automated image analysis. Further QTL mapping studies in *Z. tritici* identified QTLs affecting several important life history traits including the degree of colony melanisation, thermal adaptation, and fungicide sensitivity (Lendenmann et al., 2014, 2015, 2016).

In contrast to the challenges associated with QTL mapping, the main challenge for GWAS is to identify one (or several) suitable natural field populations. The population should exhibit heritable variation in the phenotypic traits of interest and little sub-structure due to limited gene flow or recombination. A well-chosen GWAS population is expected to have a significantly higher mapping resolution than a population used for QTL mapping (Hall et al., 2010). However, the power of GWAS can be significantly reduced by the inclusion of related individuals and population substructure, both of which can lead to spurious associations of phenotypes and genotypes and require corrections to reduce false positive associations (Connelly and Akey, 2012). Moreover, GWAS performs poorly in detecting associations caused by rare variants and when loci have only small effects on phenotypes (Korte and Farlow, 2013).

Since the establishment of GWAS (Klein et al., 2005), applications in fungi are in their infancy compared to model plant and animal systems. One of the first GWAS in fungi was in the model yeast *Saccharomyces cerevisiae* (Muller et al., 2011). High-density genotypes of 44 clinical and non-clinical strains were used to identify loci associated with virulence. In plant pathogens, GWAS has been applied to identify virulence loci in three species. Dalman et al. (2013) found significant associations for 12 SNPs distributed across seven different contigs of the *H. annosum* genome for virulence on Scots pine and Norway spruce. Gao et al. (2016) applied reduced representation sequencing for a GWAS of *Parastagonospora nodorum*. Known effector genes (*SnToxA* and *SnTox3*) could be used as positive controls to determine the mapping power of the assay (Gao et al., 2016). This study showed that RAD-GBS approaches can be used as an alternative to whole-genome sequencing. However, the marker density and the expected decay of linkage disequilibrium in the mapping population need to be carefully considered to avoid false negatives due to insufficient marker density. In a study on *F. graminearum*, Talas et al. (2016) reported GWAS associations for aggressiveness and deoxynivalenol production in a mapping population collected across Germany.

A large GWAS of the wheat pathogen *Z. tritici* identified the first avirulence effector gene associated with aggressiveness on a wheat cultivar (Hartmann et al., 2016). The GWAS included 106 sequenced strains across four populations and identified 25 distinct loci. The major locus encoded a single, small secreted protein, which was highly expressed. Population genomic analyses showed that higher aggressiveness was strongly associated with deletions of the gene, suggesting that the gene product was recognized by a cognate host protein. Hence, the gene loss was likely adaptive for the pathogen. The gene deletion was driven by a highly polymorphic cluster of transposable elements. Interestingly, the gene was not present in any of the closely related sister species suggesting that the gene has been either acquired horizontally or has evolved *de novo* (see below for a more detailed discussion of such processes).

Although GWAS applications are still scarce for fungi, the GWAS performed to date indicate significant promise. Compared to plant and animal GWAS, even relatively small mapping populations of 50–100 unrelated individuals were sufficient to robustly identify associations. Furthermore, the high-quality genomes now available for many fungi are an excellent resource for downstream validation of GWAS associations.

### The Distinction of Core and Accessory Regions in the Genome: Is it Black and White?

**FIGURE 5:**
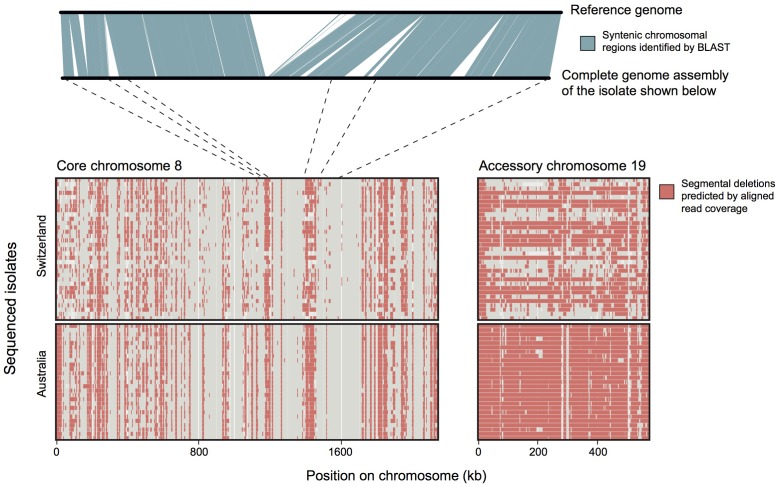
**Analyses of structural variation in populations of a fungal pathogen.** The plot above shows chromosomal synteny between two homologous chromosomes of *Zymoseptoria tritici*. Each of the two homologous chromosomes had segments lacking in the other chromosome. Such deletions in chromosomes can be also be identified using Illumina short read data aligned against a reference genome (plot below). Isolates from an Australian and a Swiss population were sequenced and reads were aligned against the reference genome. Red blocks show regions without mapped reads from a specific isolate. The dotted lines show how the above chromosomal sequence is located on chromosome 8. The accessory chromosome 19 shows longer deleted segments than the core chromosome 8. Accessory chromosome 19 is missing entirely from the Australian population and missing in about half of the Swiss isolates. Datasets shown here are from Hartmann et al. (2016) and Plissonneau et al. (2016b).

Population genomics is a powerful tool to study pathogen evolution. Many population genomics analyses are restricted to variation at SNPs, yet fungal populations often harbor high degrees of plasticity in their genomes (**Figure 5**). Genes involved in host-pathogen interactions are frequently located in rapidly evolving genomic compartments. The heterogeneity across the genome was called the “two-speed” genome and most often refers to compartments distinguished by uneven mutation rates, GC-content and gene density (Raffaele and Kamoun, 2012). For a comprehensive understanding of the factors governing effector gene evolution, considering the mechanisms that govern genome plasticity is a key step.

Genome size varies extensively among eukaryotes and is only weakly correlated with organismal complexity or the number of genes in the genome (Lynch, 2007). All but the most compact eukaryotic genomes contain genomic regions enriched in non-coding sequences. A key question on how genome structure evolves is why non-coding DNA accumulates in genomes despite the potentially high costs of mutational burden. Lynch (2007) hypothesized that the mutational cost of genome expansion is dependent on the efficiency of selection and, hence, the effective population size. Large, repeat-rich genomes are predominantly found in species with low effective population sizes. Uneven evolutionary rate across the genome is a universal feature. However, whether repeat-rich regions contribute to adaptive evolution in taxa outside of the realm of plant pathogenic fungi remains unclear.

Filamentous pathogens exhibit great flexibility in genome size. This is largely explained by the accumulation of TEs in various genomes (Raffaele and Kamoun, 2012). Some fungal pathogens such as *L. maculans* (Rouxel et al., 2011) have a genome that has expanded as a result of a massive invasion by TEs, while *Sclerotina sclerotiorum* (Amselem et al., 2011) has a smaller genome virtually devoid of TEs. Mobile elements predominantly have negative effects on their hosts and increase mutation rates. Defense mechanisms such as RIP (repeat-induced point mutation) (Selker, 1990), MIP (methylation induced pre-meiotically) (Goyon and Faugeron, 1989) and quelling (Romano and Macino, 1992) have evolved to counteract TEs expression and mobility. TEs have been shown to play a prominent role in the evolution of genome structure and pathogenicity in several fungi (Fudal et al., 2009; Manning et al., 2013; Faino et al., 2016).

As many plant pathogenic fungi have genomes expanded by TE invasions, the hypothesis that “bigger can be better” was proposed when faced with antagonistic co-evolution with a host (Raffaele and Kamoun, 2012). The “two-speed” genome concept highlights the compartmentalization into repeat-dense regions with higher mutation rates and regions with a high gene density that remains fairly conserved over evolutionary time (Raffaele and Kamoun, 2012). The “two-speed” genome was hypothesized to shelter genes encoding essential, housekeeping functions in the core genome, whilst allowing novel genes to evolve in the accessory genome (Raffaele et al., 2010; Croll and McDonald, 2012). In plant pathogenic fungi, the two-speed genome is thought to contribute to the potential to rapidly evolve virulence.

“Two-speed” genome compartments identified by distinct sets of chromosomes (e.g., core and accessory chromosomes) are the simplest to identify structurally and functionally. For example, the secreted in xylem (*SIX*) virulence genes of *F. oxysporum* f. sp. *lycopersici* are located on accessory chromosomes and the loss of these chromosomes leads to a loss of virulence toward tomato (Ma et al., 2010). In many fungi, the origin of accessory chromosomes is uncertain and may be species-specific. In some species accessory chromosomes are hypothesized to have originated *via* horizontal transfer, while in other species an origin *via* the degeneration of essential chromosomes is the most likely explanation. Interestingly, accessory chromosomes are not unique to fungi. These chromosomes, referred to as B chromosomes, have also been identified in mammals (Hayman and Martin, 1965), insects (Wilson, 1907) and plants (Longley, 1927). In several instances B chromosomes exhibit sequence homology with “core” (or A) chromosomes and are hypothesized to have a multi-chromosomal origin with sequences originating from several of the A chromosomes (Camacho et al., 2000). B chromosomes do not pair with A chromosomes during meiosis and are considered to be autonomously replicating selfish genetic elements with no apparent function.

While virulence factors are frequently found in repeat rich regions, not all accessory compartments have a clear association with the ability to cause disease. *Z. tritici* has the largest accessory chromosome complement identified thus far, consisting of eight chromosomes of various sizes (Goodwin et al., 2011). These chromosomes encode a large array of genes with largely unknown functions and low expression levels during infection (Rudd et al., 2015). Despite this, there is a correlation between the presence of certain accessory chromosomes and increased virulence (Stewart et al., 2016). During sexual crosses the accessory chromosomes were inherited more frequently than expected, suggesting that segregation distortion may serve as a mechanism to prevent loss in populations through genetic drift (Croll et al., 2013). The accessory chromosomes frequently underwent rearrangements and were present in more than one copy in some cases (Wittenberg et al., 2009; Croll et al., 2013).

Accessory chromosomes can accelerate the evolution of pathogenicity. This may happen through horizontal transfer, as has been shown for *F. solani* (Temporini and VanEtten, 2004), *F. oxysporum* (Ma et al., 2010) and *Alternaria alternata* (Akagi et al., 2009). In *F. oxysporum*, host specificity is encoded by genes on accessory chromosomes, as the transfer of chromosome 14 into a non-pathogenic isolate confers virulence on tomato (Ma et al., 2010). In *Colletotrichum* species, the causal agent of anthracnose on different hosts, evidence was found for HGT from bacteria and plants (Jaramillo et al., 2015). In a recent study, Vlaardingerbroek et al. (2016) showed that “core” regions of the genome could be transferred horizontally when accompanied by the transfer of accessory chromosomes. Although HGT is believed to have played a role in the origin of some accessory regions, the ability to be transferred horizontally does not clearly distinguish between core and accessory genomic compartments in many lineages. Overall, horizontal transfer seems to be a major contributor to the emergence of new pathogens (van der Does and Rep, 2007).

Within species, chromosome length polymorphisms are frequent among homologous “core” chromosomes, suggesting that chromosomes harbor highly polymorphic compartments (Zolan, 1995). Hence, the distinction between the regions of the genome that are evolving rapidly and regions that are more static is more blurred than previously thought. For example, both core and accessory chromosomes of *Z. tritici* are highly polymorphic and segregate segmental deletions in field populations (**Figure 5**). *L. maculans* effector genes are located in AT-rich, repetitive isochores that are arbitrarily distributed between the gene-rich, high GC content regions of the genome (Rouxel et al., 2011). Similarly, in *P. infestans* effector genes are located in gene-sparse regions that have a seemingly random distribution in the genome (Tyler et al., 2006; Haas et al., 2009; Raffaele et al., 2010).

Whether pathogens have a low-repeat one-speed genome such as *Leptosphaeria biglobosa* or *Ustilago maydis* (Kämper et al., 2006; Grandaubert et al., 2014), or a “two-speed” genome with core, and accessory regions, the general trend is that virulence genes were rarely found in conserved compartments of the genome. However, the structural distinction between the genome compartments of the “two-speed” genome is rarely a clear dichotomy: accessory and core, fast and slow. The distinction between the slowly and rapidly evolving genome compartments is likely a gradual transition in, e.g., gene density, accompanied by changes in transposons and repetitive elements content.

There is no experimental evidence that pathogens benefit from a “two-speed” genome (needless to say that such experiments may not be feasible in the first place). However, the evolutionary trade-offs involved in carrying a “two-speed” genome may well be investigated. The rapidly evolving compartment in pathogen genomes is largely controlled by transposable elements. While the activity of such selfish elements can clearly produce beneficial mutations in virulence loci, the overall spectrum of deleterious changes plays an important role in determining the selective advantage. If the rare beneficial mutation is accompanied by an overwhelming number of deleterious mutations, then evolution of such a “two-speed” genome should be counter-selected.

However, if deleterious mutations can largely be prevented, the “two-speed” genome should be strongly favored. The rapidly evolving genome compartment may be best seen as a largely selfishly governed compartment that accidentally produces benefits to the pathogen genome in which it is hosted. Such a largely non-adaptive view of the “two-speed” genome would reconcile views on the organization of genome structure across eukaryotic kingdoms.

### How Do Effector Genes Emerge in Fungal Genomes?

**FIGURE 6:**
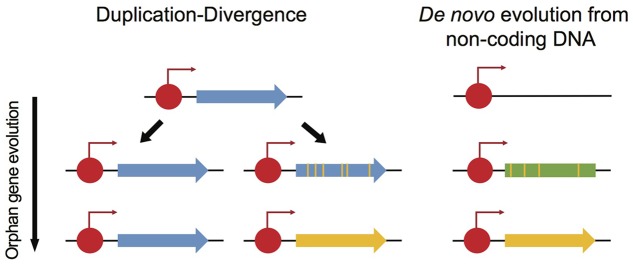
**Two models explaining the evolution of orphan genes.** Orphan genes can originate from duplication-divergence events. Following a duplication event, one copy of the ancestral gene diversified to the extent that homology is no longer detectable (yellow vs. blue gene). Red circles show transcription factor binding sites. An orphan gene can also originate *de novo* from non-coding sequences at a transcription factor binding site. Mutations in transcribed but non-coding sequences can lead to the emergence of an open reading frame (ORF).

As many fungal effector genes lack homologs in closely related species, such effector genes may best be described as orphan genes. Orphan genes are genes exclusively found in a species or in a taxonomic lineage (Tautz and Domazet-Lošo, 2011). The identification of orphan genes, or “taxonomically restricted genes,” is a major interest in many eukaryotic lineages, as orphan genes are likely playing a key role in speciation and specialization (Long et al., 2003). Two major hypotheses have been proposed to explain the emergence of orphan genes in genomes (Tautz and Domazet-Lošo, 2011). The first scenario for orphan gene evolution is called “duplication-divergence” (**Figure 6**): following a gene duplication event, one copy diverges to the extent that homology is no longer detectable and the gene simultaneously evolves a distinct function. This mechanism was originally considered as the only source of *de novo* gene evolution in genomes (Jacob, 1977). However, genes can also evolve *de novo* from non-coding DNA by fixing mutations required for a functional gene (**Figure 6**; McLysaght and Guerzoni, 2015). This mechanism requires both the evolution of an open reading frame (ORF) from non-coding DNA and functional *cis*-regulatory elements. Analysis of *de novo* gene birth from proto-genes in *Saccharomyces cerevisiae* suggested that *de novo* gene birth is likely a widespread mechanism in eukaryotic genomes (Carvunis et al., 2012), though, most the of *de novo* genes are short-lived and only a small fraction is likely to become fixed in a species (Palmieri et al., 2014).

Plant pathogenic fungi can harbor repertoires of 100s of putative effector genes (Lo Presti et al., 2015), how effector genes emerge in fungal populations remains unknown. Though, as effectors can confer a significant fitness benefit, orphan genes evolving to encode an effector protein function should be under strong selection pressure. The hypothesis of effectors being orphan *de novo* genes is challenged by the fact that effector genes are often highly up-regulated *in planta*. In contrast, *de novo* genes are characterized by their low expression as compared to core genes (Carvunis et al., 2012). Transcription of effector genes is tightly regulated and synchronized during plant colonization (Kleemann et al., 2012; Soyer et al., 2014; Palma-Guerrero et al., 2016). Hence, regulatory evolution is required to properly express a *de novo* effector gene at the critical time point of plant colonization. The transcriptional synchronization is especially important for the switch between biotrophic and necrotrophic phases in hemibiotrophic fungi.

The evolutionary trajectory of orphan effector genes needs to be put into the perspective of the co-evolution driving host-pathogen interactions. Genes encoding effector proteins that are recognized by host receptor proteins (i.e., avirulence effectors) are often rapidly lost or mutated. When this occurs, frequently lost “core” effector genes could then be mistaken as orphan *de novo* genes. The preferential localization of effector genes in gene-poor and repeat-rich regions may hold strong clues about the emergence and rapid evolution of effector genes. The evolution of effector genes is tightly linked to the activity of transposable elements (Rouxel et al., 2011; Castanera et al., 2016; Dutheil et al., 2016; Faino et al., 2016; Yoshida et al., 2016).

It is important to note that not all effector genes are orphans and some are highly conserved among fungal species. The LysM effector protein family is present in most fungal species, independently of their lifestyle (Akcapinar et al., 2015). LysM effectors interact with chitin compounds to protect fungi from plant chitinases or inhibit the recognition of chitin oligomers by plant receptors (Sánchez-Vallet et al., 2015b). The avirulence effector *Ave1* in *V. dahliae*, which triggers *Ve1*-mediated resistance in tomato, shares homologs across a broad range of species, including bacteria. *Ave1* is likely to have been acquired from plants through horizontal transfer (de Jonge et al., 2012). The *Ustilago maydis* avirulence effector Pep1 is another example of a widely conserved effector, as it is found in most smut species (Hemetsberger et al., 2015). Moreover, in some cases, despite a complete lack of sequence homology, effector proteins share strong similarities in their 3D structure. Avr1Co39, AvrPi-A, AvrPItZ in *Magnaporthe oryzae* and ToxB in *Pyrenophora tritici-repentis* completely lack sequence homology but have very similar 3D structures, which suggests that these proteins share a common function (Guillen et al., 2015).

### Is the Role of Effectors in Virulence Reflected in the Evolutionary History of Effector Genes?

Gene-for-gene model interactions trigger a strong immune response in the host, which prevents colonization by the pathogen. The deployment of major *R* genes (i.e., qualitative resistances) in crop cultivars is very effective to prevent pathogen epidemics. However, qualitative resistances exert strong selection pressures on avirulence effector loci. As a result, qualitative resistances can be rapidly overcome by pathogen populations. Hence, increasing the durability of resistance genes is a major challenge in agro-ecosystems. The cost of virulence associated with the loss of an avirulence effector is an opportunity to infer the potential durability of a qualitative resistance in fields (Leach et al., 2001), but only few fungal effectors have been functionally characterized and their exact role in virulence can be difficult to assess. First, the lack of homologs of effector genes in closely related species makes the identification of effector genes *in silico* very challenging. Second, effector genes can be functionally redundant, which leads to false negative outcomes in single-gene knockout studies.

Diverse evolutionary mechanisms allow fungal populations to escape *R* gene mediated recognition. Deletion of avirulence effector genes is a common mechanism that has been observed in many plant pathogenic fungi (Schürch et al., 2004; Gout et al., 2007). The inactivation of an avirulence gene can also result from its disruption by the insertion of a transposable element (Inami et al., 2012). The modification of the avirulence protein is another mechanism to escape host recognition. In these cases, effector gene sequences are likely to show hallmarks of positive selection (Iida et al., 2015; Poppe et al., 2015). In *Melampsora lini*, changes in the amino acid sequence of the avirulence protein AvrL567 allows to escape recognition by three distinct resistance proteins (L5, L6, and L7) (Dodds et al., 2006).

Moreover, fungi evolved complex mechanisms to escape effector-triggered immunity without any modification of the corresponding avirulence gene. In the tomato fungal pathogen *F. oxysporum*, the presence of the avirulence gene *Avr1* suppresses the recognition of both avirulence genes *Avr2* and *Avr3* (Houterman et al., 2008). Similarly, in *L. maculans*, the avirulence gene *AvrLm4-7* allows to escape the recognition of the avirulence gene *AvrLm3* (Plissonneau et al., 2016a). In both cases, the “masked” avirulence genes are expressed and the virulence function of the encoded proteins is likely to be maintained. This suggests that the loss of the “hidden” avirulence genes would impose an important fitness cost.

### Future Directions

Over the past decade, the outstanding progress in fungal genomics revolutionized our knowledge of plant pathogens. However, the progress inevitably opened numerous new questions. Despite the prediction of extensive effector gene repertoires in many fungal genomes, only a few effectors were experimentally shown to play a role in virulence. Moreover, most effector genes lack homologs in closely related species, which limits both our understanding of effector gene evolution and our ability to reliably identify effector functions *in silico.* Plant pathogen population genomics offers an excellent opportunity to determine the genetic basis of many fungal phenotypes, including virulence. In particular, techniques such as GWAS, QTL mapping and genome scans were already shown to be powerful tools to identify effector genes involved in host-specific interactions.

Most population genomics analyses are currently limited to variation at SNPs, though structural variations are also likely to play an important role in fungal genome evolution. The emergence of third-generation sequencing technologies, providing sequencing reads in excess of 10 kb at reasonable costs, is a milestone to capture the full complexity of polymorphism in fungal genomes. Complete genome assemblies revealed the important roles transposable elements play in genomic plasticity and effector gene evolution (Faino et al., 2016; Plissonneau et al., 2016b). However, our knowledge of structural variation in fungal populations is still limited. Expanding the analyses of complete genomes to the population and species scale will provide a particularly powerful approach to understand the emergence of evolutionary novelty in pathogens.

Population genomics of plant pathogens offers also an opportunity to indirectly assess the durability of major resistance genes in agricultural fields. For example, effector genes showing mostly presence/absence polymorphism in populations are likely to be dispensable for host colonization. These genes will impose either no or low costs of virulence for the pathogen. On the contrary, effector genes under strong positive selection for protein modifications or epistasis with other effectors, the fitness cost associated with virulence should be high.

## Author Contributions

All authors listed, have made substantial, direct and intellectual contribution to the work, and approved it for publication.

## Conflict of Interest Statement

The authors declare that the research was conducted in the absence of any commercial or financial relationships that could be construed as a potential conflict of interest.
